# Children’s Transport Built Environments: A Mixed Methods Study of Associations between Perceived and Objective Measures and Relationships with Parent Licence for Independent Mobility in Auckland, New Zealand

**DOI:** 10.3390/ijerph16081361

**Published:** 2019-04-16

**Authors:** Melody Smith, Rebecca Amann, Alana Cavadino, Deborah Raphael, Robin Kearns, Roger Mackett, Lisa Mackay, Penelope Carroll, Euan Forsyth, Suzanne Mavoa, Jinfeng Zhao, Erika Ikeda, Karen Witten

**Affiliations:** 1 School of Nursing, The University of Auckland, Auckland 1142, New Zealand; rama919@aucklanduni.ac.nz (R.A.); a.cavadino@auckland.ac.nz (A.C.); d.raphael@auckland.ac.nz (D.R.); jinfeng.zhao@auckland.ac.nz (J.Z.); 2 School of Environment, The University of Auckland, Auckland 1142, New Zealand; r.kearns@auckland.ac.nz (R.K.); e.forsyth@auckland.ac.nz (E.F.); 3 Department of Civil, Environmental and Geomatic Engineering, University College London, London WC1E 6BT, UK; roger.mackett@ucl.ac.uk; 4 Human Potential Centre, Auckland University of Technology, Auckland 1142, New Zealand; Lisa.mackay@aut.ac.nz (L.M.); erika.ikeda@aut.ac.nz (E.I.); 5 Social and Health Outcomes Research and Evaluation (SHORE), Massey University, Auckland 1142, New Zealand; P.A.Carroll@massey.ac.nz (P.C.); k.witten@massey.ac.nz (K.W.); 6 Melbourne School of Population and Global Health, The University of Melbourne, Melbourne 3010, Australia; suzanne.mavoa@unimelb.edu.au

**Keywords:** traffic safety, walking, cycling, infrastructure, active travel, active transport

## Abstract

Children’s independent mobility is declining internationally. Parents are the gatekeepers of children’s independent mobility. This mixed methods study investigates whether parent perceptions of the neighbourhood environment align with objective measures of the neighbourhood built environment, and how perceived and objective measures relate to parental licence for children’s independent mobility. Parents participating in the Neighbourhood for Active Kids study (*n* = 940) answered an open-ended question about what would make their neighbourhoods better for their child’s independent mobility, and reported household and child demographics. Objective measures of the neighbourhood built environment were generated using geographic information systems. Content analysis was used to classify and group parent-reported changes required to improve their neigbourhood. Parent-reported needs were then compared with objective neighbourhood built environment measures. Linear mixed modelling examined associations between parental licence for independent mobility and (1) parent neighbourhood perceptions; and (2) objectively assessed neighbourhood built environment features. Parents identified the need for safer traffic environments. No significant differences in parent reported needs were found by objectively assessed characteristics. Differences in odds of reporting needs were observed for a range of socio-demographic characteristics. Parental licence for independent mobility was only associated with a need for safer places to cycle (positive) and objectively assessed cycling infrastructure (negative) in adjusted models. Overall, the study findings indicate the importance of safer traffic environments for children’s independent mobility.

## 1. Introduction

Physical activity is fundamental to optimal growth, development, and health in children. It is recommended that children and youth aged 5–17 years should participate in at least 60 minutes of moderate-to-vigorous intensity physical activity (MVPA) daily to gain important benefits for their musculoskeletal, cardiovascular, neuromuscular, metabolic, and mental health and development [1]. Yet physical activity levels are low globally [2], including in New Zealand, where a third of children and youth are insufficiently active for health [3].

### 1.1. Children’s Independent Mobility

Children’s independent mobility can provide important opportunities for children’s physical activity accumulation. Independent mobility was first defined in the early 1990s as having the freedom to travel to destinations or engage in outdoor play without adult supervision [4]. Since that time, numerous definitions, associated metrics, and methods (e.g., parent vs. child reports) have been employed [5,6,7]. For the most part, literature has focused on parental licence for independent mobility, capturing a range of ‘licences’ to be independent outside the home environment. To a lesser extent, actualized independent mobility has also been explored, with a dominant focus on active travel (e.g., walking, scootering, or cycling) to school or neighbourhood destinations without adult accompaniment. Irrespective of the metric used, a growing evidence base has consistently demonstrated links between independent mobility and active travel to school [8], objectively-assessed physical activity [9,10,11,12], and less sedentary behaviour [12]. 

Independent mobility also plays an unparalleled role in contributing to children’s development of social, cognitive, and spatial processing skills and enhanced environmental awareness. Children who travel and play freely in their neighbourhood socialise more frequently with their peers and adults [13,14,15] and develop a sense of belonging to their community [16] which is important for developing social skills and a sense of identity. Cognitive and psychological development is promoted through spatial awareness and processing [17], learning about risk and its management [13], and engagement with the natural and physical environment [13,14,18,19].

Despite its benefits, independent mobility has been declining globally over the past several decades [4,20,21]. By way of example, in Australia, repeated cross-sectional studies indicate the proportion of children travelling home from school independently halved from 68% in 1991 to 31% in 2012 [20]. Longitudinal data on independent mobility in the New Zealand context are not available. Looking at active travel in general (irrespective of accompaniment) the average time children spent in active travel modes decreased from 130 minutes per week to 72 minutes per week in the two decades prior to 2011. Latest household travel statistics show 70% of travel time for children is spent as a car passenger, and 10% is spent in active travel modes [20]. Less than half of New Zealand youth actively travel to school [3]. Accordingly, there has been increasing interest in understanding factors related to children’s active travel in general as well as their independent mobility. 

### 1.2. Factors Associated with Children’s Independent Mobility

A socio-ecological perspective is helpful to understand the complexity of children’s independent mobility [6,22]. In particular, the systems model proposed by Badland and colleagues [23] provides a robust framework for understanding factors related to children’s independent mobility. The model encompasses policy and societal norms, as well as factors at the individual and neighbourhood level, which are described briefly below.

#### 1.2.1. Child and Household Characteristics

Parental licence for their child’s independent mobility is strongly related to children’s actualised independent mobility [24]. Parents are the ‘gatekeepers’ who determine the travel mode of their children and the degree of freedom they have to independently move in the neighbourhood [6,25]. Although children seek to influence their mobility licence [26], parents’ views are invariably stronger than children’s in determining independent mobility [27,28,29]. 

Individual factors that are associated with children moving independently in their environment include age and sex. Children who are older, especially after 12 years of age, are more likely to have licence from their parents to engage in independent mobility [9,24,30,31,32]. Many studies indicate that boys have higher rates of independent mobility compared to girls and tend to gain mobility licence earlier than girls [9,32,33]. This difference could be due to the higher parental fear of danger from strangers for girls [34,35]. 

More broadly, household and family factors such as car ownership, having older siblings [36], competing family schedules and trip chaining [37], and neighbourhood self-selection (i.e., a preference for residing in an environment that is characterized as being more or less walkable) [38,39,40] may also play important roles in actualizing children’s independent mobility. 

#### 1.2.2. Parental Neighbourhood Perceptions

Parents’ perceptions of the neighbourhood environment are associated with whether or not they grant their children independent mobility licence [41]. Parents who view their neighbourhood as safe are more inclined to encourage their children to be physically active outdoors [42]. Perceived safety of the neighbourhood environment may be an especially important indicator for parental licence for independent mobility [30,43], particularly regarding traffic safety and “stranger danger” [35,44]. If parents perceive their neighbourhoods as unsafe they are less likely to allow their children to be independent [45]. Conversely, a positive parental perception of safety from crime in the neighbourhood is associated with greater mobility licence [30,32]. “Stranger danger” has been noted as a concern for parents and has been linked to limited licence for independent mobility [9,34,35,46]. On the other hand, social interactions with neighbours [47] and social cohesion and trust [31,32,48] could contribute to creating a neighbourhood that ‘looks out for children’ and thus encourages independent mobility. 

Safety from traffic is a key priority for parents [24,49]. Numerous studies have shown that parental fear of traffic danger is associated with limiting children’s independent mobility licence [21,32,35,36,45,50,51]. Wolfe and McDonald [32] concluded that parents who had a positive view of traffic safety in their neighbourhood were more likely to grant their children licence for independent mobility. Parents who lived on busy roads [51], who had concerns about high traffic volumes [32], or who lived in less urbanised areas [35] had more traffic safety concerns and their children had less independent mobility. On the other hand, safe road crossings were associated with increased independent mobility of boys in a study of Australian children [51].

Moving beyond neighbourhood safety, a number of built environment features, as perceived by parents, have been related to independent active travel and physical activity of children [28]. Specifically, perceived accessibility to destinations, high land use mix, short distance to school, residential density, and well-maintained walking and cycling infrastructure have all been positively associated with children’s independent active travel.

#### 1.2.3. Objective Measures of the Environment

The objectively-assessed built environment has also been significantly associated with children’s independent mobility [51]. Sharmin and Kamruzzaman [52] argue that changes in children’s independent mobility are related to the built environment context (e.g., distance to destination and availability of facilities), urban structure (e.g., urban vs. suburban) or urban form alterations (e.g., intersection density, traffic safety infrastructure). However, to date the evidence remains sparse and inconsistent, thus this section also includes some relevant literature on active travel and physical activity.

Street connectivity has been positively associated with active travel [53,54] and independent mobility [55] in some studies. Conversely, there is also evidence that culs-de-sac (dead end streets) are strongly associated with independent mobility [52]. To some extent, this may be a function of the metric of independent mobility used. For example, where independent active travel is the focus, street connectivity would be expected to have a significant role in understanding independent mobility. Conversely, where independent play is of interest, prevalence of neighbourhood culs-de-sac in which to play may be of greater importance. Linked with street connectivity, accessibility to destinations such as schools, parks, and other local settings has been positively associated with active travel and independent mobility [45,54]. Villanueva and colleagues [51] concluded that, in addition to the proximity of destinations, the aesthetics and appeal of destinations are also important features of the built environment that facilitate independent mobility.

Studies in Portugal have shown that when comparing rural and urban areas, increased urbanisation leads to decreased independent mobility of children [24,35]. On the other hand, a meta-analysis by Sharmin and Kamruzzaman [52] concluded that urban–suburban residential location type, and a higher proportion of commercial and residential land use are positively associated with children’s independent mobility. To some extent these mismatches may be because these broad environmental measures are not sufficiently fine-grained or population-specific to be relevant to parental decision-making around their child’s independent mobility. The field is advancing, however, with examples being the development of new child-specific measures for walkability [56] and destination accessibility [57].

There is also some uncertainty and inconsistency about how objective built environment measures and parent perceptions of the built environment relate, especially in regard to traffic safety. Carver and colleagues [27] identified that there is vagueness as to whether the neighbourhood traffic safety perceptions of parents impact children’s physical activity more than objective road safety measures. To date, this has not been clear. Marzi and colleagues [41] concluded in their systematic review that parents’ perception of neighbourhood, including that of traffic safety, have a greater impact on children’s independent mobility than related built environmental measures. In contrast, Uys and colleagues’ study in South Africa concluded that the objective built environment was more strongly associated with out-of-school physical activity of children [58]. 

### 1.3. Study Aim and Objectives

It is likely that both objective and perceived environmental factors are important in understanding children’s independent mobility. Hence, how they relate to each other and with parental licence for independent mobility needs to be better understood to guide and inform future research and practice. The overall aim of this study was to provide new knowledge about factors associated with parental licence for children’s independent mobility. Study objectives were: (1) to identify key factors of importance from parent perspectives that would support children’s independent neighbourhood active travel, (2) to contextualise parent perceptions in relation to objectively assessed variables around the individual household address, and (3) to determine associations between parental licence for independent mobility and objective neighbourhood built environment measures and parent-reported needs.

## 2. Materials and Methods

### 2.1. Protocol and Study Context

Neighbourhoods for Active Kids was a cross-sectional study of children residing in Auckland, New Zealand. Auckland is New Zealand’s largest city with a population of 1.4 million people, a third of New Zealand’s total population [59]. The urban landscape of Auckland is comprised largely of suburban neighbourhoods, however there is a move to more compact city housing due to pressures of increasing population and lack of affordable housing [60].

The full study methods are described elsewhere [61]. In brief, addresses of all intermediate schools (junior high school, years 7–8, approximate ages 10–13) in Auckland were geocoded and child-specific measures of walkability [56] and destination accessibility [57] generated for each school. A matrix of walkability, destination accessibility, and school decile (a measure of area-level socioeconomic status [62]) was then used to select schools for invitation, with the aim of ensuring heterogeneity in these factors across the study neighbourhoods. Ensuring heterogeneity in geographic location (north, south, east, west, and central Auckland) was also prioritised. For each intermediate school, a contributing primary school (elementary school, years 1–6, approximate ages 5–11 years) was also invited to participate in the study, resulting in a primary-intermediate school dyad in each neighbourhood. 

Either Principal or Board of Trustees (governing body of school) consent for their school to participate was required before initiating participant recruitment. Research team members visited each school to explain the study protocols to teachers and students, and deliver child and parent information sheets, child assent forms, and parent/caregiver consent forms for their child and themselves to participate. Those interested in participating were asked to return the signed forms to the school within two weeks (during which time children and their families were invited to ask the research team any questions they had about the study). A signed assent form and signed parent/caregiver consent form were required for a child to participate in the study.

Researchers visited schools during school time to undertake an online participatory mapping survey to capture children’s neighbourhood use and perceptions, measure school routes and perceptions of the school route, and capture measures of food purchasing and consumption of unhealthy foods. Height, weight, and waist circumference were measured by trained researchers at this time and children were provided with an accelerometer on a belt and instructions on how to wear the units for the next seven days. Post collection of the accelerometers, a computer-aided telephone interview (CATI) was conducted with parents/caregivers of participating children. The CATI collected sociodemographic information about the child and household, parent neighbourhood perceptions, licence for their child’s independent mobility, and reports of their child’s activity and nutrition behaviours. For each school, a face-to-face (or telephone-based) semi-structured interview was conducted with the school Principal or delegate to capture contextual information about supports for active travel to school and related policies and initiatives. Objective measures of the built environment for physical activity and nutrition were generated around the home neighbourhood environment, school neighbourhood environment [63], school route [8,64], and home-school neighbourhood [65]. Measures specific to the current study are described below.

Data were collected between February 2015 and December 2016. Ethical approval to conduct the study was provided by the host institution ethics committees (AUTEC, 14/263, 3 September 2014; MUHECN 3 September 2014; UAHPEC 9 September 2014).

### 2.2. Measures

#### 2.2.1. Socio-Demographic Information

Parents reported on the biological sex (male, female) of their child and main ethnic group in the CATI survey. Ethnicity variables were adapted to combine ‘Middle Eastern/Latin America/African’ (*n* = 17) ‘other’ (*n* = 4), and ‘not stated’ (*n* = 158) due to low frequencies. School level (primary, intermediate) was recorded during the school-based data collection and used as a proxy for age. Parents were asked how many working cars they had available to them in their household. Area-level socio-economic status was determined using the NZDep2013, an index of deprivation comprising Census-derived measures of income, housing tenure, employment, educational qualifications, family structure, household crowding, access to transport, and communications at the meshblock level (smallest Census-area unit) [66]. Deciles were aggregated into categories of low deprivation (decile 1–3), medium deprivation (decile 4–7), and high deprivation (decile 8–10).

#### 2.2.2. Independent Mobility Licence

Licence for independent mobility was assessed using six items from the Policy Studies Institute study of children’s independent mobility [21]. Parents were asked whether their child was allowed to travel home from school alone, cross main roads on their own, cycle on main roads alone, go out alone after dark, and go on local public transport on their own. Response options were: always, often, sometimes, and never. Parents were also asked whether their child was usually taken or allowed to go alone to places other than school that were within walking distance. Response options were: usually goes alone, varies, and usually taken. All six items were considered in principal components analysis to generate a measure of independent mobility licence. Bartlett’s test of sphericity (*p* < 0.001), and the Kaiser–Meyer–Olkin measure of sampling adequacy (0.697) were deemed acceptable. One component was extracted which explained 32.5% of the variance. A higher score indicated higher licence for independent mobility.

#### 2.2.3. Parent-Reported Needs 

Parents were asked “What would make your neighbourhood a better place for (Child Name) to walk, bike or scooter by (Himself/Herself)?” Responses were open-ended and parents could mention more than one factor, or choose not to respond.

#### 2.2.4. Objectively-Assessed Neighbourhood Built Environment Features

Objective measures were calculated in ArcMap 10.5 (Environmental Systems Research Institute, Redlands, CA, USA) using an 800 m pedestrian street network buffer around each child participant’s residential address only. Within the pedestrian network, all motorways and state highway segments were removed to best reflect the pedestrian environment. Unless otherwise specified, spatial data were sourced from the Auckland Council, via the University of Auckland’s GeoData Hub database.

##### Ratio of High- to Low-Speed Roads

Traffic speed exposure was measured as the ratio of high speed (>60 km/hour) road length (HSRL) to low speed (<60 km/hour) road lengths (LSRL) within the neighbourhood boundary [56]. The ratio was calculated as HSRL/LSRL, with a higher ratio indicating a greater number of high-speed roads relative to low-speed roads, and thus greater exposure to vehicular traffic. 

##### Number of Signalised Crossings

Data on pedestrian crossings at controlled intersections (i.e., with a crossing light) were downloaded from Auckland Transport’s Open GIS Data platform (https://data-atgis.opendata.arcgis.com/). A spatial join between each neighbourhood buffer and the pedestrian crossing points was implemented, with the sum count of points intersecting each buffer returned.

##### Ratio of Cycle Path Lengths to Road Lengths

Data for Auckland’s cycle lane network were drawn from Auckland Transport’s Open GIS Data platform (https://data-atgis.opendata.arcgis.com/). Total cycle lane length (CLL; km) within the neighbourhood buffer was divided by all road lengths (ARL; km), regardless of speed limits to generate a ratio of CLL/ARL, where a higher ratio indicates more cycle lane availability relative to roads.

##### Pedestrian Network Connectivity (PedShed)

PedShed was measured as the ratio of reachable pedestrian network area (network buffer area; NBA) to the maximum possible area (Euclidean buffer area; EBA) within a given distance of each participant’s residence [56]. The NBA was derived using the pedestrian street network buffer, and the EBA via a Euclidian buffering of the residence point. The pedestrian network connectivity variable was calculated as NBA/EBA, with a higher ratio indicating that a greater proportion of the maximum possible distance can be reached through the pedestrian network and, therefore, a more connected pedestrian network.

### 2.3. Data Analysis

#### 2.3.1. Objective 1

Content analysis was performed on parent responses to the CATI question “What would make your neighbourhood a better place for (Child Name) to walk, bike or scooter by (Himself/Herself)?” The lead author undertook initial coding with 40% of responses to develop a coding framework for key topics and subtopics (saturation was achieved). Deborah Raphael then independently coded all responses using the framework. Finally, the lead author randomly cross-checked 20% of the full coded dataset. Any disagreements were resolved, and decisions made from this process were used to determine the final coding framework used to code the data. Descriptive statistics for whether or not a subtopic was mentioned by parents were calculated. These were summed to calculate overall frequency of key topics being reported by parents.

IBM SPSS Statistics 24 (IBM Corp, Armonk, NY, USA) was used for all descriptive and inferential analyses. From the content analysis, four topics were identified (based on ranking first and secondly on availability of related objective measures) and recalculated as binary outcomes (i.e., whether parent had reported the topic or not). 

#### 2.3.2. Objective 2

Mixed effects logistic regression modelling was employed to examine whether there were any significant differences in parent-reported needs by their corresponding objectively-assessed neighbourhood features (outlined in Table 1). All models were adjusted for school level, sex of child, child ethnicity, and deprivation, with neighbourhood included as a random effect. All ratio measures were reclassified into binary variables using the median value to classify variables as low or high [56]. A *p*-value threshold of 0.05 was used to determine statistical significance. 

#### 2.3.3. Objective 3

Differences in parental licence for independent mobility were tested for socio-demographic variables using Mann–Whitney U Tests (for sex and school level) and Kruskal–Wallis H Test (for ethnicity and deprivation). Non-parametric tests were used due to non-normal distribution of independent mobility licence. Mixed-effects linear models were used to examine relationships with parental licence for their child’s independent mobility, including the corresponding perceived and objective measures simultaneously in each model. Sex of child, school level, child ethnicity, and deprivation were all included as fixed effects and neighbourhood was included as a random effect. Interactions between objective and perceived measures were also tested. A *p*-value threshold of 0.05 was used to determine statistical significance.

## 3. Results

Overall, 19 schools across 9 neighbourhoods in Auckland participated in the study. In total, 940 parents participated in the study, providing information about their household, child, and neighbourhood perceptions. Nine cases were eliminated from the analysis due to missing data, yielding a final sample of 931 parents.

### 3.1. Objective 1: Parent-Reported Needs

Parents’ responses to the question “What would make your neighbourhood a better place for (Child Name) to walk, bike or scooter by (Himself/Herself)?” fell broadly under nine key areas as outlined in Table 2. A majority of parents (88.1%) thought there were physical and social environmental aspects that could make their neighbourhood better for their children to be able to walk, cycle, and scooter around. Three percent of parents made positive comments about their neighbourhoods such as:

“I feel that this area is very community focused and it feels quite safe and feels quite child friendly.”

Over half of parents (50.3%) mentioned a need for a safer transport environment (less, slower, and safer traffic; and having safe places to cross, cycle, and walk), with the greatest need being less, slower, and safer traffic (19.9%). Specifically, parents consistently noted a need for the following:

Slower and safer drivers, for example: “If people keep to the speed limit it would make me feel safer”

Less traffic: “The road traffic is busy and too hard for the kids to use.”

more traffic-calming infrastructure: “More speed bumps and more signs near the schools so people can see them and slow down.”

Lower speed limits: “Lower speed limits or speed bumps around the area as the people tend to speed down the road and there are a lot of kids in the area that play near the roads.”

Signage such as “kids around” to slow traffic and encourage safe driver behaviour: “More caution signs on [X] Road where we live as the traffic whizzes through…More caution signs in the hope that people will pay attention to these signs.”

Safer places to cross: “More pedestrian crossings on the roads as cars speed around the area”

Safer places to cycle: “I’d like to see a dedicated cycle lanes attached to the footpaths that link up to the school.”

Safer places to walk: “Decent footpaths as we have no footpaths on our side of the road and so this is a big deterrent. Also cycle ways would be great as in the bays here we have narrow winding roads.”

The social environment of the neighbourhood was also significant for parents. Twelve percent of parents reported that safety from others (in particular reduced sense of “stranger danger”, increased community surveillance, reduced drug and gang related crimes, fewer roaming dogs and less bullying from youth) would make their neighbourhood better. A more connected community and having more people ‘out and about’ in their neighbourhood was considered important by 3.5% of the parents, for example:

“I guess if we all get together as a neighbourhood and get to know each other very well then we could all look out for each other’s kids and we would get to trust each other.”

### 3.2. Quantitative Modelling

#### 3.2.1. Descriptive Characteristics

Sociodemographic characteristics of children were relatively evenly distributed with the exception of ethnicity, where a majority were of New Zealand European/Pākehā/Other European ethnicity (42.3%) (Table 3). Parental licence for independent mobility scores were positively skewed, ranging from 3.30 to 10.72 with a mean of 5.28 (*SD* = 1.67). Boys had significantly higher licence for independent mobility than girls (*p* < 0.001). Similarly, older children had significantly higher licence for independent mobility than their younger peers (*p* < 0.001). There was a significant difference in licence for independent mobility between ethnic groups with Pacific children having lower independent mobility than other groups (*p* = 0.001). Almost all parents (98.4%) reported having at least one working car available in their household, with 78% reporting availability of two or more cars. Descriptive information for the objectively assessed neighbourhood environmental features is provided in Table 4.

#### 3.2.2. Objective 2: Differences in Objectively-Assessed Neighbourhood Features by Parent-Reported Needs

Given the parental focus on transport-related topics (and the availability of corresponding objective data), the following four variables were considered in further analyses related to objective measures and parental licence for independent mobility: less, slower, and safer traffic; more and safer crossings; safer and designated cycle lanes; and more and better walking paths.

Results from mixed effects logistic regression models testing for differences in odds of reporting a need by objectively-assessed neighbourhood built environment and socio-demographic characteristics are presented in Table 5. Taking socio-demographic factors and neighbourhood into account, no statistically significant differences in parent reported needs were observed between those residing in a more or less supportive neighbourhood (objectively assessed). Differences in odds of reporting needs were observed for a range of socio-demographic characteristics. Parents of primary school-aged children had significantly higher odds of reporting a need for less, slower, and safer traffic (OR 1.54, *p* = 0.014), and significantly lower odds of reporting a need for safer and designated cycle lanes (OR 0.48, *p* = 0.003). Compared with those of New Zealand/other European ethnicity, parents of children of Pacific ethnicity had significantly lower odds of reporting needing less, slower, and safer traffic; more and safer crossings; and safer and designated cycle lanes (ORs 0.06 to 0.36, all *p* < 0.01). All ethnic groups were significantly less likely to report a need for safer and designated cycle lanes compared with the reference group (NZ European/pākehā/other European; ORs 0.06 to 0.51, all *p* < 0.05). Differences in deprivation were only observed for reporting a need for more and better walking paths, with those from medium or high deprivation areas significantly less likely to report this need compared with those residing in areas of lower deprivation. 

#### 3.2.3. Objective 3: Relationships between Parental Licence for Independent Mobility and Objective Neighbourhood Features and Parent-Reported Needs 

Results from linear mixed models of relationships between objectively-assessed neighbourhood features and parent-reported needs and parental licence for independent mobility are presented in Table 6. No significant interactions between objective neighbourhood environment variables and parent reported needs were observed. In the fully adjusted models, both parent-reported need for safe cycling infrastructure (positive) and objectively-assessed cycle infrastructure (negative) were significantly related to parental licence for independent mobility. Children of parents who thought more dedicated and safer cycle lanes in their neighbourhood would make it better for their children to walk, bike, and scooter, had 0.56 higher parental independent mobility licence score on average, compared to parents who did not mention safer places to cycle (*p* = 0.001). Parent licence for their child’s independent mobility was an average of 0.46 lower for those residing in areas with a higher ratio of cycle path to road length compared with those who lived in areas with a lower ratio of cycle path to road length.

## 4. Discussion

The aim of this study was to understand factors related to parental licence for their child’s independent mobility in a large sample of parents of children aged 8–13 years residing in Auckland, New Zealand. A sequential approach was taken using mixed methods. Firstly, data derived from parent interviews were used to determine factors that parents perceived would make their neighbourhood better for their child to be independently mobile. Secondly, objective neighbourhood built environment variables were generated and examined in relation to parent reported needs. Finally, associations between parental licence for their child’s independent mobility were determined with key objective and perceived neighbourhood variables. Key findings were that: (1) parents identified a need for safer traffic environments for their child’s independent mobility; (2) no significant differences were observed between objective neighbourhood built environment measures and parents’ reported neighbourhood needs; and (3) parental licence for their child’s independent mobility was positively associated with parent perceptions that dedicated and safer places to bike were needed in their neighbourhood and negatively associated with residing in an area with a higher ratio of cycle path to road lengths. These findings are discussed in detail below and contextualized within independent mobility literature, supplemented by evidence for children’s active travel where relevant.

### 4.1. Objective 1: Parents’ Perceptions on What Would Make Their Neighbourhood Better for Independent Mobility

Content analysis of parents’ answers to the question “what would make their neighbourhoods better for their child to walk, cycle and scooter in?” showed that a majority of the parents believed aspects of their neighbourhood could be improved. A key finding was that parents identified a safer traffic environment as a major need for their children’s independent mobility (50.3% of parents overall). Particularly, less, slower, and safer traffic (19.9%); more pedestrian crossings (13.4%); more designated cycle lanes (9.8%); and more and better walking paths (7.2%) were considered important aspects.

Traffic safety is consistently a parental concern and limiting factor for children’s independent mobility [35,67,68] and active travel in general. The current study findings aligned closely with the body of work in this field, for example in the early focus group research of Ahlport and colleagues [69], parents identified high traffic volume, dangerous drivers, and busy intersections as specific barriers to their child’s active travel. 

For the most part, findings related to perceptions of transport infrastructure also aligned with previous research. For example, parental concern for lack of pedestrian crossings was related to less active travel in a study of Australian children [29] and presence of pedestrian crossings was associated with more independent travel in a later study [49]. On the contrary, Evers and colleagues [70] found that traffic lights, marked crossings, and bump-out crossings did not influence parents’ concern for their children crossing the road. However, these results were based on whether the parent would let an eight-year-old child cross the road unsupervised, and the age of the child may have been a bigger influence on parental concerns than the crossing infrastructure. In the current study, almost 5% of parents noted that age of their child was the key driver of their child’s independent mobility licence, rather than the neighbourhood environment. There is likely an age and stage threshold below which infrastructure and neighbourhood perceptions are irrelevant in determining whether a child is allowed to be independently mobile.

Parents also highlighted the need for safe places to walk and cycle as key environmental considerations. These findings add to a small evidence base about the importance of availability and condition (e.g., uneven surfaces, obstacles, overgrown vegetation) of walking paths for children’s independent mobility [70]. As well as providing a safe place to walk, the availability of walking paths may be important as this can reduce the need to cross roads to reach a destination [69]. In an adult sample, half of the respondents believed that lack of sidewalks in their neighbourhood was a problem and was a barrier to them being physically active [71]. Parents also noted the condition of walking paths, specifically that paths should be well maintained and wide enough for children to safely walk and cycle away from the road. 

Safe places to cycle were also identified by parents as key infrastructural features that would create a supportive environment for children to be independently mobile. Specifically, parents mentioned the need for cycle lanes that are dedicated and separated from the road. This preference aligns with earlier research reporting on parental views on the importance of cycling infrastructure [28,72]. More specifically, cycle lanes that are separated from road traffic are perceived vital to cycling safety by parents. When considering relative importance of varying traffic infrastructure, one study reported that parents’ perceived physical separation of child cyclists and the road was more important than a lower speed limit [73]. 

Safety from others was also raised by 12% of parents, with a focus on concerns about “stranger danger” (5.4% of parents) and a need for greater community surveillance (4.6%). To a lesser extent, criminal and gang activity, roaming dogs, other “undesirables”, and bullying were also identified as factors limiting children’s independent mobility. Concern about safety from strangers is an established barrier to parents granting licence for independent mobility to their child [9,34,46] as is the fear of crime [32]. Increased perceived social cohesion is associated with greater independent licence as it may support the perception of a safer neighbourhood for children [31]. However, Foster and colleagues [34] found no association between informal social control and decreased fear of strangers, indicating that the these perceptions are multifaced and may not be alleviated simply by a more supportive neighbourhood community.

### 4.2. Objective 2: Perceived and Objective Aspects of the Neighbourhood Transport Environment

In fully adjusted models, no statistically significant differences were observed in parent reported needs by different levels of the objective measures. In other words, no significant association was found between the objectively-assessed neighbourhood features and reporting a need. This finding was somewhat expected, considering that while the perceived and objective measures were theorized to relate to each other, they were not assessing the same environmental dimensions exactly (as detailed in Table 1). Additionally, a growing body of evidence has shown that adult perceptions do not always align with objective measures of the neighbourhood environment [71,74,75]. Rothman and colleagues [76] concluded that there are some differences between parents’ perception and objective measures of traffic safety along school routes. However, some specific measures such as road crossings were related to lower parental perceived traffic danger. McGinn and colleagues [71] also found no agreement between perceived fast and heavy traffic and objectively measured speed and traffic volume. It is likely that both approaches provide valuable insights, and the triangulation of multiple data sources is optimal to gain a comprehensive understanding of environmental features of importance. Indeed, past research examining objective and perceived measures of the neighbourhood environment in relation to independent mobility has shown mixed results. Marzi and colleagues [41] concluded from their systematic review that parental perceptions of traffic have a greater influence in determining children’s independent mobility compared with objective measures of the physical environment. Looking across the lifespan, perceptions were stronger indicators than objective measures for physical activity in preschoolers [77], active travel to school in children [78], and adults walking in their neighbourhood [79]. On the contrary, there is evidence that adults’ physical activity is supported by urban design features regardless of their perception of the neighbourhood [80]. In adult populations, both McGinn and colleagues [71] and Lee and Dean [81] concluded that it may be necessary that both perceptions and objective measures of the neighbourhood environment are used to understand the complexity of the relationship between the built environment, walkability, and physical activity.

After adjusting for all other factors, differences in parents’ perceived needs by socio-demographic characteristics were observed, including child’s school level (less, slower, and safer traffic; cycling infrastructure), ethnicity (all variables except walking infrastructure), and area level deprivation (walking infrastructure only). These novel findings highlight the link between socio-demographic factors and neighbourhood perceptions as well as aiding understanding relationships between perceived and objective environments. Parents of younger children had 1.5 times the odds of reporting a need for less, slower, and safer traffic compared with those of older children. This implies the age of a child influences parent traffic safety perceptions, more so than the actual traffic environment. Compared with older children, a higher traffic safety threshold may be required for younger children in order for parents to perceive their environment as being safe. Conversely, parents of younger children were half as likely to report a need for safer and designated cycling infrastructure. It is possible this is due to lower levels of cycling in younger children compared with older children in this study (data not reported here) and thus cycling infrastructure being less relevant for these parents. In a similar manner, all ethnic groups were significantly less likely to report a need for safer and designated cycle lanes compared with parents of children who identified as being of New Zealand European/Pākehā/Other European ethnicity. National travel survey data suggest significantly higher rates in those of New Zealand European ethnicity compared with other ethnic groups (and low rates of cycling overall) [82]. Parents of children of Pacific ethnicity also had significantly lower odds of reporting needing less, slower, and safer traffic; more and safer crossings. It is unclear why these differences existed—further work examining ethnic differences in neighbourhood perceptions would be worthwhile. 

Differences in deprivation were only observed for reporting a need for more and better walking paths, with those from higher deprivation areas significantly less likely to report this need compared with those residing in higher deprivation. It is possible this is a reflection of the New Zealand context, where positive relationships have been found consistently between walkability elements (e.g., destination accessibility) and deprivation [83].

### 4.3. Objective 3: Factors Associated with Parent Licence for Independent Mobility

Independent mobility licence was significantly related to both parent-reported need for safer cycling infrastructure and objectively-assessed cycle infrastructure. Children had a higher independent mobility licence when their parents mentioned that their neighbourhood needed more dedicated and safer cycle lanes, compared with children of parents who did not mention cycling infrastructure. This finding aligns with earlier work by Timperio and colleagues [29] indicating it is possible that parents are more aware of their traffic environment when they grant their children more independent mobility licence. Specifically, if children cycle in their neighbourhood, parents may notice the lack of cycle lanes available for their children, while in comparison parents who grant low mobility licence may not identify the need for more cycling lanes. Neighbourhood self-selection [39] may play a role in understanding parent perceptions. It is possible that parents who prefer neighbourhoods with safer transport infrastructure have chosen to live in neighbourhoods that align with this preference, yet their own personal preferences are still not being met sufficiently.

In terms of objectively measured cycling infrastructure, children with higher parental licence for independent mobility lived in areas with lower cycle lane availability. This finding could be due to parents allowing their children to be independently mobile, but more so for walking rather than cycling. Indeed, Moran and colleagues [53] concluded that walking is a more common form of independent mobility than cycling, and that different built environmental features are associated with each type of active travel mode. 

No other significant associations with independent mobility were found. Inconsistent findings have been observed in earlier literature with regard to links between parental perceptions and children’s independent mobility. Traffic safety perceptions of parents was positively associated with adolescent active travel [84] and independent mobility in girls [72]. Conversely, Santos and colleagues [43] found no association with traffic safety and independent mobility. Perceived poor availability of road crossings has been associated with decreased independent mobility for boys [51] and decreased active travel in children, generally [85,86]. In other studies, parent perceptions of available walking paths was a predictor for independent mobility [43] and active travel [28]. 

A recent meta-analysis identified a number of built environment features related to children’s independent mobility (operationalized as independent mobility time, independent mobility to destinations, territorial range, or licence for independent mobility) [52]. Positive associations were found for proportion of residential land, proportion of commercial land, residential location type, and dead-end streets. Negative associations were observed for vehicular street width, road density, street connectivity, proportion of major roads, land use mix, availability of recreational facilities, residential density, and distance to destinations. Situating the current study findings with the extant literature, it is clear that understanding determinants of children’s independent mobility is a complex challenge. Factors exist across the socio-ecological spectrum [23], and the role of parenting practices, beliefs, and perceptions in this context cannot be underestimated [87]. Nuances may also exist, whereby differences in relationships might be expected by neighbourhood social and built environment contexts and spatio-temporal factors may also play a role. For example, earlier work with Auckland children showed that active travel was associated with destination accessibility on weekdays only, and differential relationships between activity and built environment characteristics were also observed between weekend days and weekdays [54].

## 5. Strengths and Limitations

This study was cross-sectional and conducted in one city in New Zealand only. Findings cannot be generalized to other environments, and causality cannot be inferred. Parental licence for their child’s independent mobility was employed in the current study to align with the majority of literature in this field. However, it is important to recognise this measure does not necessarily encompass all facets of children’s independent mobility (e.g., time, range, destinations) and that children may also report their independence differently from their parents. Had different measures of independent mobility been used in the current study, it is possible alternative findings might have been identified. It is also worth noting that broader family and household characteristics may play an important role in children’s independent mobility, including having older siblings, household car ownership, competing family schedules, and neighbourhood self-selection. The latter three variables were not measured in this examination. The almost universal access to a working car in this study hindered any ability to detect the effect of not having access to a car on children’s independent mobility. The pervasiveness of the car is consistent with national data. New Zealand has one of the highest rates of car ownership internationally and rates have continued to rise in recent years [88]. It is possible these broader factors are more likely to be linked to children’s actualized independent mobility, rather than parental licence for independent mobility, and further work in this area is needed. It is also possible that other parent psychological factors and experiences may have contributed to understanding children’s independent mobility and parent neighbourhood perceptions, however these were not examined in the current investigation. For example, factors such as anxiety, values around child independence, incidences or ‘near misses’, and local narratives about past incidents related to child independent mobility could all play a role in how a parent perceives their environment and thus impact the independence granted to their child. 

No agreed upon approach for determining neighbourhood buffers for children’s independent mobility exists. Previous examinations in Neighbourhoods for Active Kids have used a range of study-specific buffers to determine environments of importance including 800 m around schools only for outdoor advertising [63], 800 m around home plus school (less any overlap) for children’s body size [65], and school route only using 80 m on each side of the street centre line for active school travel [64]. The “home only” buffer was determined for the current study to align with the study’s focus on parent perceptions about their neighbourhood and their child’s independence in the neighbourhood. Not all children lived within close proximity to school, so including a school buffer to characterize the objective built environment and examine this in relation to parent perceptions would have reduced sensitivity and specificity. Previous research with Auckland children supported a 1000 m buffer to capture levels of MVPA [54]. Conversely, a Canadian study showed 500 m was optimal to measure associations with girls’ MVPA, and 800 m was best for boys’ MVPA [89]. An 800 m buffer was chosen in the current study as a pragmatic approach to capture sufficient environmental features that might be ‘front of mind’ for parents when considering their neighbourhood and their child’s independent mobility, and to allow comparability with previous research [51,54,89]. Future research would benefit from the use of more individually-centred measures such as activity spaces [90].

## 6. Conclusions

Through the use of mixed methods with a large sample of parents, this study provides a comprehensive examination of children’s independent mobility licence, parent neighbourhood perceptions, and objectively assessed neighbourhood built environments. For the first time, objective pedestrian crossing and cycle lane availability has been examined in relation to children’s independent mobility, contributing to improving sensitivity and specificity in neighbourhood built environment measures. Novel findings on relationships between socio-demographic characteristics and parent neighbourhood perceptions can help interpret research exploring differences between perceived and objectively-assessed neighbourhood features. Further research is needed to explore the complex relationships between parent psychological characteristics, family and household context, perceived and objective neighbourhood environments and children’s actualized independent mobility and licence for independent mobility across a range of settings. Novel findings demonstrate the importance of measuring both perceived and objective characteristics, and exploring why these do not always align. Overall, this novel study demonstrates the importance of providing safer traffic environments, particularly safer places to cycle, for children’s independent mobility.

## Figures and Tables

**Table 1:** Parent reported need and corresponding objective measure of the neighbourhood built environment.

Parent-Reported Needs	Objectively-Assessed Neighbourhood Features	Key Considerations Regarding Making Direct Comparisons
Less, slower, and safer traffic	Ratio of high to low speed roads	Objective measure provides an estimate only of traffic safety based on road hierarchy (and does not account for actual driver behavior irrespective of regulatory environment); parent-reported traffic safety needs included a broad range of strategies to reduce speeds and volume of traffic
More and safer crossings	Number of signalised crossings	Objective measure only takes signalled crossings into account; parent-reported needs included comments about non-signalled crossings
Safer and designated cycle lanes	Ratio of cycle path lengths to road lengths	Objective measure is limited to data available for the cycle network; parent-reported needs included bike paths and also the quality of places to cycle safely
More and better walking paths	PedShed	Objective measure provides an indication of the relative prevalence of places to walk only; parent-reported needs noted additional considerations such as wider and better-maintained footpaths

**Table 2:** Descriptive statistics for key topics derived from parent responses to the question “What would make your neighbourhood a better place for (Child Name) to walk, bike or scooter by (Himself/Herself)?” (*n* = 931).

Topic and Subtopics	*n* ^a^	% ^a^
**Safety from traffic: Less, slower, and safer traffic**	185	19.9
Less busy traffic	65	7.0
Slower speeds	56	6.0
Traffic calming infrastructure (e.g., humps)	38	4.1
Lowering speed limits	37	4.0
Reducing dangerous driving	34	3.7
Improving traffic safety in general	19	2.0
Signage to slow traffic (e.g., “kids around”, “slow down”)	6	0.6
**Safety from traffic: More and safer crossings**	125	13.4
More and safer pedestrian crossings	121	13.0
Lights at pedestrian crossings	4	0.4
Supervised crossings	1	0.1
**Safety from traffic: Safer and designated cycle lanes**	91	9.8
Cycle lanes—designated, away from road, on footpaths	66	7.1
Bike tracks and paths	26	2.8
**Safety from traffic: More and better walking paths**	67	7.2
**Safety from others**	112	12.0
Reduced “stranger danger”	50	5.4
Community surveillance	43	4.6
Reduced crime (drugs and gang activity)	19	2.0
Fewer roaming dogs	12	1.3
Reduced perceived danger from others especially youth	9	1.0
Less bullying	4	0.4
**More and better destinations**	37	4.0
More destinations in the neighbourhood	23	2.5
More and better facilities at the destinations	16	1.7
**Better social environment**	33	3.5
More connected community	25	2.7
More children/people out and about	9	1.0
**Others**	232	24.9
Better street lighting	56	6.0
Child too young	45	4.8
Nothing	38	4.1
Positive Comments	31	3.3
Less hilly	14	1.5
Safer neighbourhood	12	1.3
Other	10	1.1
More public transport and school buses	9	1.0
Fewer cars parked on street	8	0.9
Better general infrastructure	8	0.9
More walking school buses (adult accompanying group of children to school)	7	0.8
Better upkeep of public spaces	6	0.6
Better visibility of the streets	4	0.4
Improved connectivity	3	0.3
Uncodeable	3	0.3

^a^ Data are presented for the number and percentage of parents who noted these topics and subtopics. Note: *n* and % of topics do not equate to the total of all the subtopics due to some parents mentioning more than one subtopic in one topic.

**Table 3:** Socio-demographic characteristics of children and their licence for independent mobility (*n* = 931).

Socio-Demographic Variables	*n*	%	Licence for Independent Mobility	
			Mean (SD)	*p*-Value
**Sex**				<0.001 ^c^
Male	469	50.4	5.65 (1.73)	
Female	462	49.6	5.00 (1.67)	
**School level**				<0.001 ^c^
Primary (years 5–6)	486	52.2	4.58 (1.32)	
Intermediate (years 7–8)	445	47.8	6.04 (1.69)	
**Ethnicity**				0.001 ^d^
New Zealand European/Pākehā/Other European	394	42.3	5.41 (1.61)	
Māori	112	12.0	5.49 (1.67)	
Pacific	125	13.4	4.94 (1.73)	
Asian	120	12.9	5.04 (1.62)	
MELAA ^a^/Other/Not stated	180	19.3	5.25 (1.77)	
**Area-level deprivation ^b^**				0.106 ^d^
Low Deprivation (decile 1–3)	357	38.3	5.36 (1.67)	
Medium Deprivation (decile 4–7)	324	34.8	5.31 (1.65)	
High Deprivation (decile 8–10)	250	26.9	5.13 (1.71)	

^a^ MELAA = Middle Eastern, Latin American, or African; ^b^ NZDep13 score calculated for each individual household at the meshblock level; ^c^ *p*-value from Mann-Whitney U Test; ^d^ *p*-value from Kruskal-Wallis H Test.

**Table 4:** Descriptive information for objectively-assessed neighbourhood features (*n* = 931).

Objectively-Assessed Neighbourhood Features ^a^	Minimum	Maximum	Mean (SD)
Ratio of high to low speed roads	0.00	1.00	0.37 (0.11)
Number of signalised crossings	0.00	7.00	1.01 (1.40)
Ratio of cycle paths to road lengths	0.00	0.74	0.12 (0.13)
PedShed ^b^	0.01	0.63	0.32 (0.11)

^a^ Variable corresponding to parent-reported need; derived within the 800 m street network individual neighbourhood buffer using Geographic Information Systems; ^b^ Ratio of reachable pedestrian network area to the maximum possible area; a higher ratio indicates a more connected pedestrian network.

**Table 5:** Differences in parent-reported needs by objectively-assessed neighbourhood built environment characteristics and socio-demographic factors (*n* = 931).

Parent-Reported Need ^a^ *n* (%) of Parents Reporting Need	Less, Slower, and Safer Traffic *n* = 185 (19.9%)		More and Safer Crossings *n* = 125 (13.4%)		Safer and Designated Cycle Lanes *n* = 91 (9.8%)		More and Better Walking Paths *n* = 67 (7.2%)	
*Objective Measure*	*Ratio of High to Low Speed Roads*		*Number of Signalised Crossings*		*Ratio of Cycle Path to Road Lengths*		*PedShed ^b^*	
	OR (95% CI) ^c^	*p*-Value	OR (95% CI) ^c^	*p*-Value	OR (95% CI) ^c^	*p*-Value	OR (95% CI) ^c^	*p*-Value
**Objective measure** (higher vs. lower) *^d^*								
Lower	Reference		Reference		Reference		Reference	
Higher	0.79 (0.54, 1.14)	0.208	0.90 (0.72, 1.11)	0.314	1.02 (0.56, 1.87)	0.944	0.77 (0.34, 1.73)	0.527
**Sex**								
Male	Reference		Reference		Reference		Reference	
Female	0.93 (0.67, 1.31)	0.693	0.73 (0.49, 1.09)	0.125	0.78 (0.49, 1.24)	0.296	0.87 (0.47, 1.60)	0.527
**School level**								
Intermediate (years 7–8)	Reference		Reference		Reference		Reference	
Primary (years 5–6)	1.54 (1.09, 2.19)	0.014	1.01 (0.67, 1.52)	0.980	0.48 (0.29, 0.77)	0.003	1.41 (0.66, 3.00)	0.372
**Ethnicity**								
NZ European/Pākehā/Other European	Reference		Reference		Reference		Reference	
Māori	0.78 (0.43, 1.44)	0.432	0.60 (0.27, 1.34)	0.214	0.12 (0.03, 0.52)	0.004	0.30 (0.06, 1.53)	0.147
Pacific	0.36 (0.17, 0.76)	0.008	0.13 (0.03, 0.59)	0.008	0.06 (0.01, 0.50)	0.009	0.29 (0.05, 1.55)	0.148
Asian	0.61 (0.34, 1.10)	0.103	0.48 (0.22, 1.03)	0.058	0.28 (0.11, 0.75)	0.012	0.37 (0.07, 1.82)	0.220
MELAA ^a^/Other/Not stated	0.85 (0.51, 1.42)	0.543	0.93 (0.49, 1.75)	0.816	0.51 (0.26, 1.00)	0.049	2.01 (0.73, 5.52)	0.174
**Area-level deprivation ^b^**								
Low	Reference		Reference		Reference		Reference	
Medium	1.08 (0.72, 1.61)	0.708	0.83 (0.53, 1.28)	0.396	0.84 (0.51, 1.39)	0.504	0.15 (0.07, 0.33)	<0.001
High	1.08 (0.60, 1.96)	0.796	0.50 (0.22, 1.18)	0.113	0.40 (0.15, 1.08)	0.071	0.22 (0.07, 0.67)	0.008

^a^ Variable corresponding to parent-reported need; derived within the 800 m street network individual neighbourhood buffer using Geographic Information Systems; ^b^ Measure of walking path availability; area of network buffer/area of radial buffer; ^c^ Estimates from mixed effects logistic regression with random intercept for neighbourhood; ^d^ Objective measures are included in each model as binary variables to compare those with ‘higher’ and ‘lower’ objective measures, defined as being above or below the median value for each.

**Table 6:** Mutually adjusted association of parent reported needs and related objective neighbourhood features with children’s licence for independent mobility (*n* = 931).

Neighbourhood Variables in Model	Comparison	Estimate (95% CI) ^a^	*p*-Value
Ratio of high to low speed roads ^b^	Higher vs. lower	−0.05 (−0.26, 0.17)	0.682
Less, slower, and safer traffic ^c^	Parent reported vs. didn’t report need	−0.03 (−0.2, 0.21)	0.811
Number of signalised crossings ^b^	Per additional crossing	−0.04 (−0.15, 0.06)	0.421
More and safer crossings ^c^	Parent reported vs. didn’t report need	0.01 (−0.27, 0.29)	0.944
Ratio of cycle path to road lengths ^b^	Higher vs. lower	−0.46 (−0.71, −0.20)	0.001
Safer and designated cycle lanes ^c^	Parent reported vs. didn’t report need	0.56 (0.24, 0.88)	0.001
PedShed ^b,d^	Higher vs. lower	0.20 (−0.01, 0.41)	0.058
More and better walking paths ^c^	Parent reported vs. didn’t report need	−0.14 (−0.55, 0.27)	0.506

^a^ Linear mixed models with random intercept for neighbourhood to account for neighbourhood clustering, including the objectively-assessed neighbourhood feature and the related parent-reported need, and adjusted for sex of child, school year level, ethnicity, and meshblock-level deprivation; ^b^ Objectively assessed variable corresponding to parent-reported need; derived within the 800 m street network individual neighbourhood buffer using Geographic Information Systems; ^c^ Parent-reported need; ^d^ Measure of walking path availability; area of network buffer/area of radial buffer.
